# Immune Microenvironment: New Insight for Familial Adenomatous Polyposis

**DOI:** 10.3389/fonc.2021.570241

**Published:** 2021-02-08

**Authors:** Jun Yang, Zhengqi Wen, Wenliang Li, Xianghua Sun, Junrui Ma, Xueke She, Hongbin Zhang, Changling Tu, Guoqiang Wang, Depei Huang, Xudong Shen, Jian Dong, Hushan Zhang

**Affiliations:** ^1^Department of Oncology, First Affiliated Hospital of Kunming Medical University, Kunming, China; ^2^Department of Cadre Recuperation, The First Affiliated Hospital of Kunming Medical University, Kunming, China; ^3^Department of Nursing, Shanghai University of Traditional Chinese Medicine, Shanghai, China; ^4^The Medical Department, 3D Medicines Inc., Shanghai, China; ^5^Department of Medical Oncology, The Third Affiliated Hospital of Kunming Medical University, Yunan Cancer Hospital, Kunming, China

**Keywords:** FAP, colorectal cancer, immune microenvironment, immune cells, cytokines

## Abstract

Currently, the main treatment for familial adenomatous polyposis (FAP) is surgery, however, surgery is far from ideal as there are many complications such as uncontrollable bowel movements, pouch inflammation, anastomotic stricture, and secondary fibroids. Therefore, it is necessary to further expand the understanding of FAP and develop new treatments for FAP. The immune microenvironment including immune cells and cytokines, plays an important role in FAP and the progression of FAP to adenocarcinoma, thus it may be a promising treatment for FAP. In the current review, we summarized the recent progress in the immune microenvironment of FAP.

## Introduction

Familial adenomatous polyposis (FAP) is an autosomal dominantly inherited disease characterized by tens to thousands of adenomas in the colorectum. Polyps usually begin to develop during childhood, and increase in size and number until adolescence. Approximately 50% of FAP patients develop adenomas by the age of 15, which increases to 95% by the age of 35. Attenuated FAP (AFAP) is a phenotypically milder form of FAP, characterized by the presence of < 100 polyps and a later onset of colorectal cancer (CRC). FAP accounts for less than 1% of colorectal cancer (CRC) cases and has an incidence at birth of about 1/8,300 in China (1).

The germline mutation of the adenomatous polyposis coli (*APC*) gene accounts for most classic cases of FAP, however, in other patients presenting similar phenotypes, particularly similar to AFAP, no *APC* mutation was identified. These are associated with biallelic mutations in the mutY homolog (*MUTYH*) gene and are known as *MUTYH* associated polyposis (MAP). APC plays a critical role in several cellular processes, such as tumorigenesis suppression, cell development by downregulating the Wnt pathway, actin and microtubule networks suppression, chromosome segregation and cell adhesion, and migration, thereby, mutation in *APC* may initiate tumorigenesis in the colon and rectum (2). Evidence of Wnt signaling pathway activation and loss of function of the tumor regulator APC have been shown in colorectal cancers, even in several other cancers such as lung cancer, breast cancer, and HCC, and it is associated with tumor recurrence (3). However, the Wnt pathway cross talks with the Sonic hedgehog and Notch pathways, which set significant challenges in targeting this pathway; to date no drugs have been approved to target this pathway.

*Apc^Min/+^* murine which carries a heterozygous mutation of *APC* is usually used as an experimental animal model for FAP research. This kind of murine can develop into approximately 30 small intestinal polyps, and these polyps can occasionally progress to invasive adenocarcinoma (4). This animal model is widely used to investigate both FAP clinical therapy and pathogenesis, for example, sulindac used in therapy of patients diagnosed with FAP (5) and the treatment of FAP patients with the COX-2 inhibitor celecoxib (6, 7).

Although most FAP can be detected and diagnosed before the development of CRC, FAP will progress to CRC if not identified and treated in time. The likelihood of FAP progressing to adenomas rises from approximate 50% to 95% along with age from 15 to 35 years, with CRC often occurring at a mean age of 39 years old (8). Prophylactic proctocolectomy or colectomy is recommended for patients with known classical FAP, and surgery is the current preferred treatment for patients with FAP symptoms according to the ACG guidelines (9). Colectomy with IRA and proctocolectomy with IPAA are the two main surgical options, and both have their merits and shortcomings (10, 11). While after surgery, there are some complications such as hemorrhage, acute pelvic sepsis, portal vein thrombus in the early postoperative period, chronic pelvic sepsis, small bowel obstruction pouch dysfunction, and pouchitis (12–14). Moreover, most adult FAP patients will progress to malignant tumors if they are not diagnosed and treated early enough (10). Therefore, it is reasonable to discern the meaningful mechanisms associated with the occurrence and development of the disease, here we try to comprehensively and systematically understand this disease from a perspective of immunity, based on this, some new strategies for the treatment of FAP may emerge, such as immunotherapy targeting various immune cells and immune factors like cytokine, chemokine, etc. Certainly, it is indispensable to understand more about the immune microenvironment (IME).

Undoubtedly, the IME is a very important and complex system associated with many diseases, especially many aspects of tumorigenesis. It seems illogically to consider IME as a simple foe or friend. Lots of components should be evaluated separately, such as various cells types (endothelial cells, fibroblasts, immune cells, *etc*.), extracellular components (cytokines, growth factors, chemokines, *etc*.), and some key molecules expressed on/in immune/tumor cells (PD-1/PD-L1, CTLA-4, etc.) (15, 16). IME is involved in disease progression and even affects the response to therapies, therefore, the regulation of the IME may improve the efficacy of therapies (17, 18).

To fully understand FAP development and progression, more attention should be paid to the IME of FAP, including the various roles of immune cells, cytokines, and chemokines secreted during the progression of FAP.

## Immune Cells

Various immune cells are major components of the FAP immune microenvironment, such as T cells, B cells, natural killer (NK) cells, macrophages, neutrophils, and epithelial cells. So far, although exploration on the FAP immune microenvironment is not comprehensive, certain excavations have been performed. By comparing the differences between FAP patients and normal individuals, or non-polypoid tissues in FAP, researchers have found that there is an increase of the lymph follicles in the terminal ileum (19, 20). Furthermore, researchers have investigated whether human colon polyps or adenomas show different lymphoid cell subsets. Compared to the normal area or normal control mucosa, CD4^+^ and IgG^+^ cells have been found significantly increased in the polyps (21). Altogether these findings implicate the involvement of immunological reactions in FAP occurrence and progression. Here we review the major immune cells related with the progression of FAP.

### Versatility of T Cells in Immune Microenvironment of FAP

**Figure 1:**
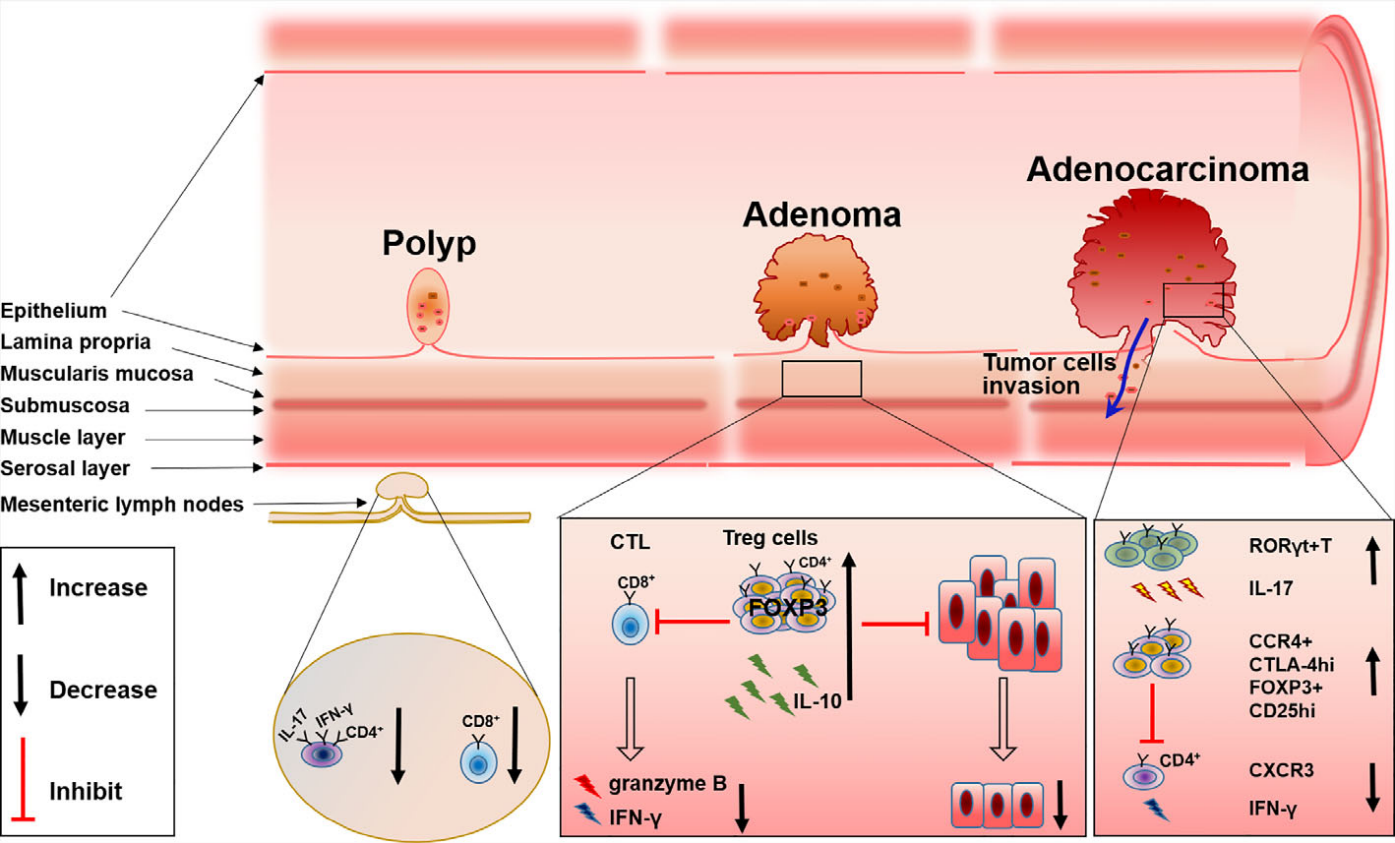
The changes of T cell subsets during FAP progress from polyp to adenoma and to adenocarcinoma.

T cells are categorized according to TCR and function, for example CD4^+^ T cells, CD8^+^T cells, or cytolytic T lymphocytes (CTL), helper T cells (Th cell), and regulatory T cells (Treg) (22). Lots of studies have shown that all of these T cell subsets participate in the immune microenvironment and work together to regulate disease progression including FAP (**Figure 1**).

#### CD8^+^ and CD4^+^ T Cells Associated With FAP

The recruitment and accumulation of CD8^+^ T cells and the regulation of CD4^+^ T cells could be used to control the development and progression of FAP. Initiation of the CTL response requires the generation of antigen peptide by proteasome and the presentation of antigen peptide by MHC-I molecules to CD8^+^ T cells, delivering a primary signal to activate antigen-specific CTL. While MHC-II molecules present extracellular antigen peptides to CD4^+^ T cells (23, 24). An imbalance of T cells in the colon will disrupt intestinal homeostasis and consequently induce the dysfunction of intestinal tumor immune surveillance, which may be one of the causes for FAP progression. This has been proven by research in *Apc^Min/+^* mice. By comparing wild-type (Apc^+^/^+^) and *Apc^Min/+^* mice, the author found that IFN-γ^+^IL-17^+^ double-positive CD4^+^ cells were decreased in the mesenteric lymph nodes and Peyer’s patches of *Apc^Min/+^* mice; the level of CD8^+^ T cells and their production of IFN-γ and granzyme B also changed. Further evidence suggested that there was a relationship between these changes in T cells and FAP progression, that compared with the adoptive transfer of splenocytes isolated from WT mice into chemically induced CRC immunodeficient mice and the adoptive transfer of splenocytes from Apc^Min/+^ mice into a CRC mice model resulted in the inability to prevent epithelial dysplasia (25). These results emphasized the necessity of a normal T cell level and function for the control of FAP progression, and evidence has shown that the recruitment and accumulation of T cells relies on the mast cell-derived leukotriene B4 (LTB4), and mice lacking the LTB4 receptor displayed accelerated disease progression and were more susceptible to intestinal tumor-induced mortality (26).

In addition, CD4^+^ T cells are also found to be involved in polyps. The number of CD4^+^ T cells is increased significantly in polyps of patients with FAP (21). Immunofluorescence experiments also support these results as conventional CD4^+^ T cells within adenomas are usually luminally clustered instead of distributed throughout the lamina propria, while CD8^+^ T cells are virtually absent from adenoma tissue (27). These studies displayed the involvement of both CD4^+^ and CD8^+^ T cells in the progression of FAP, however, the definite role and the specific mechanism of these cells in the progression of FAP are still ambiguous, deeper exploration should be performed in such a direction.

#### Role of Regulatory T Cells (Tregs) in FAP

Tumor-infiltrating regulatory T (Treg) cells, known as suppressive factors of effective tumor immunity, are also indispensable for preventing autoimmunity (28). Therefore, how these cells can be specifically targeted for normalizing tumor immunity without inducing autoimmunity is now gaining importance. By inhibiting both inflammatory cells and tumor-infiltrating lymphocytes (TILs), Tregs have also been shown to be associated with FAP. Firstly, the intrinsic connection between inflammation and cancer progression in the intestine has been well established (29), and the inhibitory function of inflammation by Tregs has also been repeatedly documented (30). Because APC may affect the differentiation of Tregs in the intestinal lamina propria, Tregs can develop into distinct phenotypes with a reduced ability to control the detrimental inflammation in the pre-cancerous intestine in FAP. Therefore, a dramatic reduction in IL-10 production by Tregs in the pre-cancerous intestine of *Apc^Min/+^* mice leads to inflammatory pathology and the growth of intestinal neoplasms (31). RORγt^+^Foxp3^+^ Tregs are considered to be involved with this phenomenon. There are a majority of colon Tregs that express both Foxp3 and RORγt, which reside in the lamina propria of the small intestine and express IL-10 instead of IL-17 (32). Researchers found that APC regulated TCR signaling, which caused a reduction of RORγt^+^Foxp3^+^ Tregs and impaired the expression of IL-10 in the pre-cancerous intestine of *Apc^Min/+^*murine (33). Although others have reported that intratumoral Tregs can convert to RORγt^+^T cells and produce IL-17 (34), all these results indicate the reduction of IL-10 accompanied with the alteration of Tregs in the tumor tissue of FAP.

Besides the alteration of Tregs differentiation, the accumulation of Tregs in colorectal tumors both in humans and in *Apc^Min/+^* mice has been widely investigated (27, 35). Tregs have an inhibitory effect on TILs which comprise of NK cells, cytotoxic CD8^+^ T cells, and CD4^+^ helper T cells, while TILs usually act as an important player in the defense against tumor growth (36). Analysis of lymphocytes isolated from unaffected patients and the tumor mucosa of patients with colon adenocarcinoma reveals that, Tregs are increased in tumors and exhibit characteristic higher expression of CTLA-4 and CCR4 (35), which probably diminishes the ability of TILs to effectively attack tumor cells. Furthermore, others reported that CD25^+^ Tregs in IME of *Apc^Min/+^* mice are increased by cyclooxygenase-2(COX-2), the inhibitor of COX-2 can reverse this phenomenon (37), and the depletion or reduction of CD25^+^ Treg cells will significantly enhance the response to PD-1 inhibition (38). These results may provide a new option for FAP or CRC therapy. At the same time, depletion of Tregs will recruit CXCR3^+^ T cells into an intestinal tumor through the production of chemokines CXCL9 and CXCL10, and the increase of IFN-γ mRNA expression (39), which further supports Tregs as a possible treatment target. However, some others hold the opposite view that CD4^+^CD25^+^ lymphocytes in *Apc^Min/+^* mice reduce the multiplicity of epithelial adenomas. Downregulation of COX-2 results in a decrease in CD4^+^CD25^+^ Tregs and tumor regression (40) (**Figure 1**).

### Various Roles of Macrophages in FAP Immune Microenvironment

**Figure 2:**
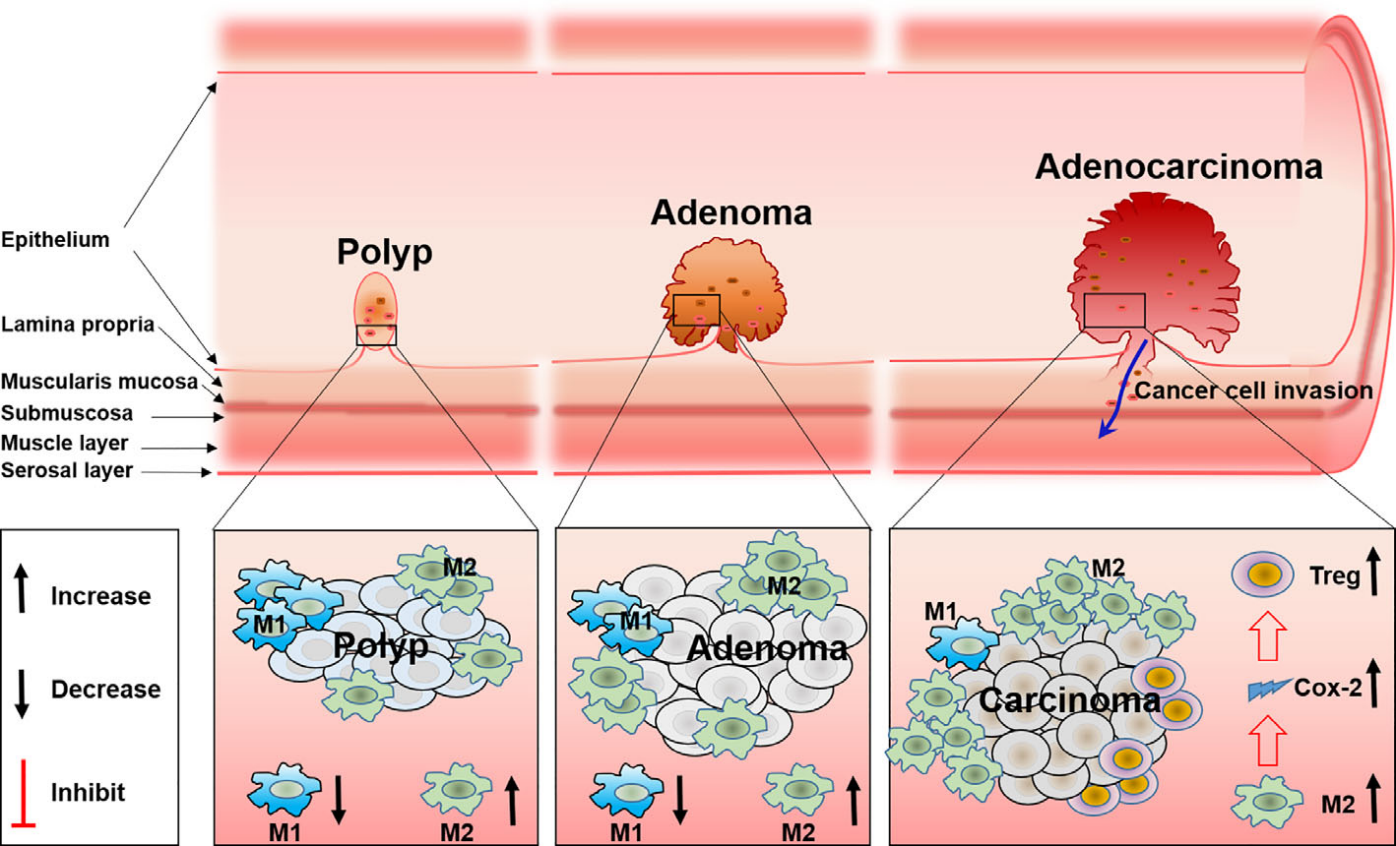
The role and the polarization of tumor-associated macrophages during FAP progress from polyp to adenoma and to adenocarcinoma.

Tumor-associated macrophages (TAM) are one of the main components in the tumor microenvironment. TAM are classified into two different subsets, M1 and M2 macrophages, according to their function and phenotype. It is well known that a higher density of M2 macrophages is closely related with worse tumor clinical prognosis, while this is opposite for M1 (41). In addition, TAM have functional plasticity and can alter their phenotype and activation status in response to the microenvironment, like polarization from M1 to M2, and the reduction of M1-related cytokines, will increase M2-related cytokines (42, 43). Present evidence has shown that TAM especially M2 macrophages are involved in the development and progression of FAP. Accompanied with the reduction of M2 macrophage polarization, the total number and size of polyps are reduced correspondingly in a FAP mice model (44). The same phenomenon has also been verified in other studies (45, 46). These results may relate the progression of polyps with the infiltration of M2 macrophages. COX-2 derived from macrophages may be the mechanism for FAP progression, in detail, analogous to human colorectal polyp growth and malignant progression. Increased stromal macrophages and COX-2 would drive *Apc^Min/+^* mouse tumor progression (47–49). Furthermore, COX-2 positive stromal macrophages in human colorectal adenomas are proven to be associated with the increase of angiogenesis, which is a possible mechanism for the progression of polyps and adenomas promoted by macrophages (49). Therefore, this confirmed macrophage and COX-2 that expressed within intestinal adenomas may serve as a putative target for anti-colorectal cancer. And indeed, it was documented that the inhibitor of COX-2 can reduce the size and number of intestinal adenomas in both FAP patients and a murine model, and the polarization of TAM from M2 to M1 induced by the COX-2 inhibitor was the mechanism (50). Although previous studies have revealed part of the function of TAM in FAP, it still remains unclear how TAM polarization and the accompanied changes of cytokines regulate the migration, activation, and distribution of other immune cells such as T cells, DC cells, or NK cells (**Figure 2**).

### NK Cells in Microenvironment of FAP

It is necessary to explore whether NK cells participate in the progression of FAP and how they participate. Natural killer cells, usually defined as CD3^-^CD56^+^ cells in humans, are distinguished as the CD56^bright^ and CD56^dim^ subsets. NK cells are presumed to be important effectors in cancer immune surveillance due to its spontaneous killing activity (51). Approximately 90% of peripheral blood and spleen NK cells are part of the CD56^dim^CD16^+^ subset with cytotoxic function (52, 53), while most NK cells in the lymph nodes and tonsils are part of the CD56^bright^CD16^+^ subset which have immune regulation properties due to the potential ability of cytokine production (54). NK cells and their kill activities in the peripheral blood of 14 patients with FAP were studied 30 years ago, while results showed that there was no change in the number or activities of NK cells (55). A similar conclusion was also found in another research paper, no significant difference was found in the number of HNK-1^+^ cells among the epithelium, the lamina propria, and the lymph follicles in FAP patients compared to those of normal large bowel mucosa (56). These studies displayed that there was no involvement of the selective impairment of NK activities and changes in the number of NK cells in peripheral blood and gut mucosa in FAP patients. It suggests that the development and progression of adenomas in FAP patients may not be attributable to abnormalities in NK cells. However, other researchers recently found that *Apc^Min/+^* mice, in addition to intestinal tumorigenesis, showed atrophy of lymph nodes, and depletion of the NK cell population in 100- and 120-day-old animals, and it seemed to be associated with the altered bone marrow microenvironment in the model of FAP. This abnormality only started at around 80 days of age, while with normal cell development before that age (57). This may account for the contradiction related to the previous research, and denote that NK cells may also unavoidably participate in immune surveillance during the progression of tumors in FAP patients. Obviously, the role of NK cells in FAP disease is very controversial and little is known about the expression of NK cells effector molecules during FAP progression, such as adhesion molecules, Fas ligand (FasL), tumor necrosis factor (TNF)-related apoptosis-inducing ligand (TRAIL), NKp44, granzymes, as well as cytokine production (58). Therefore, wider and deeper explorations are urgently needed to unveil both the cell cytotoxic and regulatory function of NK cells.

### More Immune Cells Involved in the Microenvironment of FAP

Several other immune cells including B cells, mast cells, and neutrophils, which are also involved in the progression of FAP, are expounded here from changes of number and function. Exploration for the role of B cells in FAP is insufficient, but B cells are indeed involved in the progression of FAP according to present data. In addition to T cells, B cells are another lymphocyte that play a major role in acquired immunity. B cells are essential in maintaining immune homeostasis for the reason that B cells are multi-functional cells, such as they directly defend against tumors or pathogens through the secretion of antibodies, and induce the killing activity of NK cells through antibody-dependent cell-mediated cytotoxicity (ADCC), in addition, B cells also function as an important immune regulator by means of secretion of cytokines (59). B cell numbers were significantly decreased in adenoma tissue in a mouse model of FAP (27), the decrease in cell number can be explained by cell apoptosis, migration, and cell development. In the mouse model of FAP, results showed that, at 100 days old, *Apc^Min/+^* animals exhibited a markedly reduced number of pro- and pre-B cells suggesting failure to renew the early B cell population (57). This may account for previous research in some aspects. However, little is known about which subsets of B cells were involved, due to the fact that several subsets can be classified from B cells, such as regulatory B cells, effector B cells, or B1 and B2 B cells, according to their different function and phenotype (60). Moreover, B cells are extensively demonstrated to be closely related with the regulation of inflammation and tumor progression in the gut. Through establishing dextran sulphate sodium (DSS)-induced colitis and azoxymethane (AOM) plus DSS-induced colorectal carcinogenesis in a murine model, previous research revealed that CD11b^+^ B cells (61), IgG^+^ B cells (62), and IgA^+^ B cells (63) play an important role in intestinal inflammation and tumors. Speculated by these, various subsets of B cells may also be involved in different stages or different aspects during the progression of FAP from polyps to adenoma and to adenocarcinoma. Yet little is known and more work is needed to answer these questions.

In addition, mast cells are very important in the control and defense of FAP progression. For the reason that mast cells are found with widespread distribution and respond to changes in their environment by communicating with a variety of other cells especially other immune cells, mast cells as a crucial proponent in both adaptive and innate immunity has gained increased prominence (64, 65). Through CCR2 and CCR5, mast cells are recruited into the tumor microenvironment of FAP disease, and increase CD8^+^T cell infiltration due to leukotriene B4 (LTB4) which is synthesized by mast cells (26). According to the context, mast cells have a marked influence on the local microenvironment of FAP, however, only communication with T cells has been revealed so far.

Similar to that of TAM, tumor associated neutrophils (TAN) can also be classified into two different subsets, known as N1 and N2 neutrophils, based on their anti- or pro-tumor properties (66). Some evidence has shown that along with the growth of intestinal polyps, the N2 neutrophil numbers are increased in the spleen, blood, and MLN of APC^Min/+^ mice at the age of 12 weeks, and reach a peak at 16 weeks when the development of polyps was maximal. The research concludes the role of N2 neutrophil in spontaneous intestinal tumorigenesis (67).

## Cytokines

**Figure 3:**
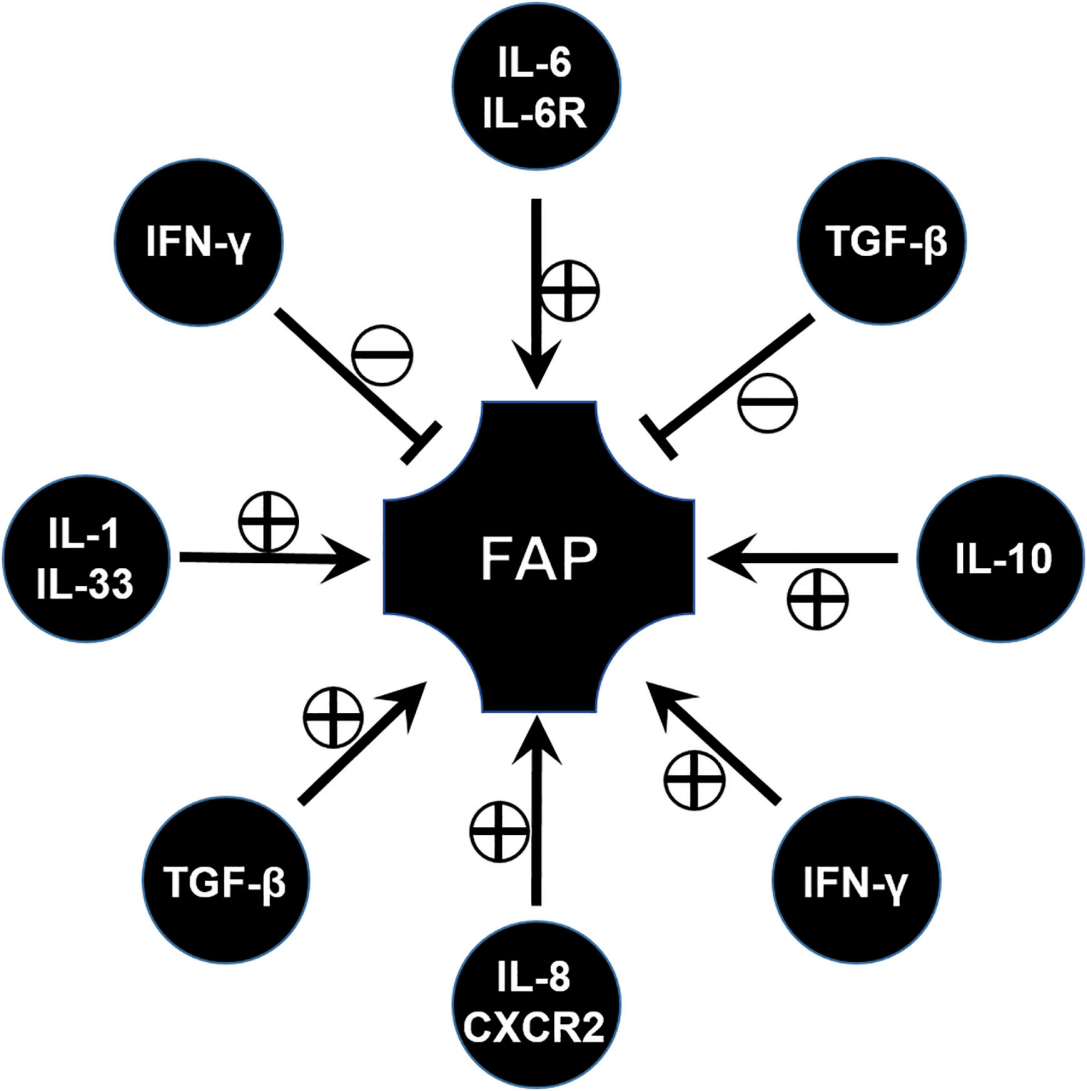
The role of various cytokines involved in the progression of FAP disease.

Cytokines are indispensably involved in the immune microenvironment, and various cytokines associated with FAP have been widely investigated during the past few decades (**Figure 3**).

### Pathogenic Role of IL-6 in FAP

IL-6 is usually considered as a pro-inflammatory factor, but in fact it is a pleiotropic cytokine that modulates a variety of physiological responses and cellular procession. For example, IL-6 affects cell proliferation, differentiation, and apoptosis through the activation of genes (68). However, only the pathogenic role of IL-6 has been studied so far. Similarly, IL-6 is found to be involved in a FAP model. Circulating IL-6 plays a role in the regulation of tumor burden and participates in the onset of adipose and skeletal muscle wasting in the *Apc^Min/+^* mouse (69). Consistent with this, blocking IL-6 signal through the anti-IL-6 receptor antibody leads to a significant decrease of the number and the diameter of intestinal polyps (70). Similar results have been validated in clinical studies which show that high levels of circulating IL-6 are associated with the presence of colorectal adenomas (71). From this aspect, IL-6 and its signaling pathway can be considered as a potential target of FAP immunotherapy (**Figure 3**).

### Pathogenic Role of IL-8 in FAP

Interleukin-8 (CXCL8) is originally known as a chemokine which can act with CXCR1/2 and by this way recruit inflammatory leukocytes (72). It upregulates IL-8 expression in intestinal tumor tissues and is associated with tumor progression, metastasis, and poor prognosis (73). High levels of IL-8 have been observed in FAP tissue, both in adenomatous polyps and adenocarcinomas (74). In vitro experiment results have shown that IL-8 can promote the adenoma–carcinoma transition from FAP (75) (**Figure 3**).

From this aspect, IL-8 and its receptor can be considered as a target to exploit drugs. For example, research found that palmatine significantly inhibited a lipopolysaccharide-nduced increase in cytokine interleukin (IL)-8, and decreased the number of tumors in an *Apc^Min/+^* mice model (76). The therapeutic potential of targeting the interleukin (IL)-8/CXCR2 pathway for FAP was also investigated through the inhibition of CXCR2 (77) (**Figure 3**).

### Bidirectional Function of IFN in FAP

IFN-γ is known as a factor for recruiting and activating monocytes and plays important roles in inducing the expression of other cytokines. IFN-γ signaling is documented with both pro- and anti-tumor activities. One study has reported the increase in expression of IFN-γ in the ileal pouches of FAP patients (78). However, FAP has been demonstrated to have low pro-inflammatory cytokine expression in some research papers (79), and some others reported that no differences in IFN-γ expression and STAT-1 activation were observed in FAP patients when compared to control individuals (80). This seems to be contradictory. Until recently, the role of IFN/STAT signaling in FAP was demonstrated through comprehensive gene expression analysis. The IFN/STAT pathway contributes to the tumorigenesis and drug response in FAP due to crosstalk between RAS signaling and IFN/STAT signaling (81). This implied IFN/STAT signaling can be applied as a potential therapeutic target for FAP. However, in a FAP murine model, lack of an IFN-γ receptor will lead to tumor progression and invasive adenocarcinomas (82), which means IFN-γ has an anti-tumor factor, and enough IFN-γ signaling is important for maintaining a tumor-prohibitive microenvironment. From this aspect, more evidence should be acquired to reveal and confirm the role of IFN-γ in FAP (83) (**Figure 3**).

### Bidirectional Role of TGF in FAP

Transforming growth factor β (TGF-β) is a well-known regulator with bifunctionality which either inhibits or stimulates diverse cell events, such as cell proliferation, differentiation, apoptosis, inflammatory responses, and tumor progression (84). As deeper research continued, TGF-β signaling was found to play context-dependent roles in FAP or CRC. Previous works have proved canonical SMAD-mediated TGF-β signaling as a colon tumor suppressor (85), similar to the function of IL-10 in FAP described in the section on Treg. While other evidence implicated TGF-β as a key pathway in the metastatic progression of colon cancer (86), by means of using APC mutant mice, results revealed that inhibiting TGF-β will lead to lethal inflammatory disease and invasive colon cancer, which implies that the application of the TGF-β inhibitor for FAP or CRC therapy is worth further investigation (87) (**Figure 3**).

### The Function of Other Cytokines in FAP

IL-33 is a pro-inflammatory cytokine belonging to the IL-1 cytokine family, which is primarily expressed by fibroblasts, epithelial cells, and endothelial cells (88, 89). Consistent with the increase of IL-1α and IL-1β (76), IL-33 is increased in epithelial cells in specimens of FAP patients and in *Apc^Min/+^* mice. In addition, blockade or knocking out of the IL-1 receptor can suppress tumor progression, which is verified by *Apc^Min/+^/ Sigirr-/-* mice. SIGIRR, defined as a single immunoglobulin IL-1 receptor-related (SIGIRR) molecule, is a negative regulator of IL-1R signaling (90). This implies that the IL-1 family, especially IL-33, could be used as a potential target for the control of CRC and FAP (91).

In addition, some other cytokines involved in the progression of FAP have also been explored. For example, TNF-α levels are higher in FAP than normal controls (92). These results may show a new direction for therapy in FAP, however, more attention should be paid to these potential targets so that these hypotheses can be verified (**Figure 3**).

## Comparison of the Microenvironment Between FAP and Sporadic CRC

Compared with CRC, IME of FAP may be regarded as part of the cancer immunoediting process during tumorigenesis and progression. Although insufficient evidence about IME of FAP has accumulated, particularly, most of these data were gained from research in animal models, it can be inferred that there must be certain connections and differences between CRC and FAP. It would be of great interest and meaning to figure out these connections and differences. Among them, there was relatively more evidence on the available analysis of T cells and the comparison between FAP and sporadic CRC. In both FAP and CRC patients, increased numbers of CD4^+^Foxp3^+^ Tregs have been shown in tumor-draining lymph nodes and tumor sites; the function of Treg cells remains controversial in both FAP and CRC (93). This was explained by the existence of two distinct Treg cell subsets with opposite functional roles in CRC: the classical Tregs displaying high expression of FOXP3, and the low FOXP3 Treg cells with inflammatory properties (94). While except for the only report on RORγt^+^Foxp3^+^ Tregs, the main attention was put on classic Tregs with immune suppressive activity. Few others were investigated and confirmed about the specific subsets of Treg cells in FAP. According to the assessment of T cell infiltration in the tumor, especially the density of CD3^+^ and CD8^+^ T cells in the tumor, CRC can be divided into high, medium, and low immune score tumors, depending on prognosis (95). It was frequently documented that the prognosis of CRC was associated with many factors such as gene mutation and status of MMR (96). Colorectal cancer with a high mutation burden usually develops due to a defect in the DNA mismatch repair system (dMMR), which can be inherited (Lynch syndrome) or acquired in sporadic cases. In addition, the immunogenic character of CRC may be associated with dMMR, that is, a pronounced lymphocytic infiltration is a hallmark of dMMR colorectal cancers, and genetic instability influences the composition of the cancer IME and determines the clinical outcome (97). However, this is only a partial explanation, the large majority of CRC are proficient MMR (pMMR) and present with low to moderate mutation burden. Therefore, other aspects should be under consideration, such as the types of gene mutation. In addition, it was found that cancer-specific T cells infiltrating IME have a specific phenotype, such as the expression of CD103 and CD39 (98, 99).

## Role of Chemoprevention Drugs in the Treatment of FAP

‘‘Chemoprevention’’ trials have been spawned by the idea of reducing polyp burden and delaying surgical intervention. Sulindac, a nonsteroidal anti-inflammatory drug (NSAID), is a commonly employed chemo-preventive drug and the most extensively studied agent in FAP. Celecoxib, rofecoxib, and aspirin, etc. have also been studied in FAP (100). The activity of these drugs to reduce polyp burden has also been extensively confirmed, even combination therapy was also explored in FAP, such as the use of sulindac and erlotinib (101). But the mechanism was mainly focused on inhibiting cyclooxygenase (COX), the key enzyme that converts arachidonic acid into prostaglandins and other eicosanoids. It is generally known that prostaglandins play a role in the adenoma–carcinoma sequence by inhibiting apoptosis, affecting cell adhesion, and promoting angiogenesis. However, evidence on the immune regulation of prostaglandins and COX-2 have accumulated over time (102). COX and prostaglandins can upregulate the expression of Th2 cytokines, while decrease the anti-tumor Th1 cytokines in immune cells, and enhance the function of immunosuppressive factors such as Treg cells and MDSCs (103, 104). Therefore, NSAID regulates immunity by inhibiting COX-2 and prostaglandins, which may be another mechanism to control the burden of polyps in FAP. In addition, NSAIDs may have anti-tumor effects independent of COX-2 inhibition, such as the regulation of TGF-β (105). In summary, based on the research evidence and application of NSAID in FAP, as well as the potential immunomodulatory effects of these drugs, it further highlights that the IME plays an important role in the occurrence and progression of FAP.

## Future Perspective

To our knowledge, FAP is a well-described inherited syndrome, characteristics of genotype, diagnosis, surveillance, and treatment strategies especially surgery treatment for familial adenomatous polyposis have been well established, however, the mechanism of progression from polyp to adenoma and to adenocarcinoma is still not clear enough, and FAP is still a risk factor for CRC. The immunotherapy of cancer including using cytokines, adoptive cell transfer therapy, active vaccination, and immune checkpoint blockades has gained some success in solid cancers, and it may also play an important role in the control of FAP and CRC, which is based on the well-known immune pattern and immune microenvironment of these diseases. For example, immune cells such as T cell subsets were closely related to the progress of FAP to CRC as described above. Specifically and precisely regulating the infiltration of these cells subsets into the tumor microenvironment can be considered as a potential target and direction to control FAP disease progression. In addition, the regulation of macrophage polarization and infiltration into tumor tissue can both directly facilitate immunotherapy and indirectly affect the expression of COX-2, which play very important roles in FAP progression. Both these immune cellular and molecular components of the IME are closely related to development and progression of FAP, for this reason, better understanding of IME will result in more potential mechanisms being figured out and being applied to practice in FAP therapy and control. In addition, as a member of the Wnt signaling pathway, APC is tightly related with phosphorylation and proteasomal degradation of β-catenin, and plays a role in the regulation of cell proliferation (106). Obviously, targeting APC and other members of the Wnt signaling pathway has long been considered a very important target for tumor therapy. Interestingly, the Wnt pathway may also be related with immunotherapy. In HCC, Wnt/CTNNB1 mutations are characteristic of a ‘cold tumor’, and it may predict resistance to immune checkpoint inhibitors (107). For FAP patients, it is also worth exploring whether the status of APC and other Wnt pathway members is related to different TME types, and whether it is responsible for the efficacy of immunotherapy in FAP patients.

## Conclusion

The importance and complications of the immune microenvironment in tumor surveillance and regulation have been widely documented and are still being investigated. FAP is characterized with the appearance of polyps at an early stage, adenoma appearance at a later age, which finally progresses into malignant adenocarcinoma, after which almost all patients will develop colorectal cancer (CRC) if they are not identified and treated at an early stage. To some extent, the progression of FAP disease can be considered the typical malignant process of tumors, gradually changing from inflammation to tumorigenesis, from benign to malignant, therefore, an investigation of the immune components during the progression of FAP, both in patients and an Apc^Min/+^ murine model, can unveil the complete background of the tumor immune microenvironment. However, exploration for this direction is far from enough, for example, specific function and real-time status of diverse immune cells during the process of FAP progression are still ambiguous. For these reasons, more attention should be put into this field. A better understanding of the immune microenvironment of FAP will lead to more found mechanisms and more potential targets for immune therapy.

## Author Contributions

JD, JY, ZW, WL, and XHS put forward the content of the paper. HSZ wrote the manuscript. XKS, HBZ, XDS, DH, CT, JM, and HSZ reviewed the literature and clinical data. HSZ prepared figures. HSZ conceived the framework of this review article, provided insights, and edited the manuscript. XKS and GW revised the manuscript. All authors contributed to the article and approved the submitted version.

## Funding

This work was supported by the National Natural Science Foundation of China (No. 81660472 and No. 81760511) and the Yunnan Applied Basic Research Projects-Union Foundation (NO. 2018FE001-142 and No. 2019FE001-073).

## Conflict of Interest

Authors HSZ, XKS, GW, XS, and DH are employed by the company 3D Medicines Inc.

The remaining authors declare that the research was conducted in the absence of any commercial or financial relationships that could be construed as a potential conflict of interest.

## Abbreviations

ADCC, Antibody-dependent cell-mediated cytotoxicity;APC, Adenomatous polyposis coli;COX-2, Cyclooxygenase-2; CRC, Colorectal cancer; CTL, Cytolytic T lymphocyte; FAP, Familial adenomatous polyposis; IME, Immune microenvironment; MAP, *MUTYH* associated polyposis; NSAID, Nonsteroidal anti-inflammatory drugs; TAM, Tumor associated macrophage; TAN, Tumor associated neutrophil; Treg, Regulatory T cells.
